# Epigenetic inhibition of Wnt pathway suppresses osteogenic differentiation of BMSCs during osteoporosis

**DOI:** 10.1038/s41419-017-0231-0

**Published:** 2018-02-07

**Authors:** Huan Jing, Xiaoxia Su, Bo Gao, Yi Shuai, Ji Chen, Zhihong Deng, Li Liao, Yan Jin

**Affiliations:** 1 State Key Laboratory of Military Stomatology & National Clinical Research Center for Oral Diseases & Shaanxi International Joint Research Center for Oral Diseases, Center for Tissue Engineering, School of Stomatology, The Fourth Military Medical University, Xi’an, Shaanxi 710032 China; 2 Xi’an Institute of Tissue Engineering and Regenerative Medicine, Xi’an, Shaanxi 710032 China; 3 Key Laboratory of Shaanxi Province for Craniofacial Precision Medicine Research, College of Stomatology, Xi’an Jiaotong University, Xi’an, Shaanxi 710004 China; 4 Department of Orthopaedic Surgery. Xijing Hospital, Fourth Military Medical University, Xi’an, Shaanxi 710032 China; 5 Department of Oral Implantology, School of Stomatology, State Key Laboratory of Military Stomatology, The Fourth Military Medical University, Xi’an, Shanxi 710032 China; 6 Department of Otolaryngology, Xijing Hospital, Fourth Military Medical University, Xi’an, Shaanxi 710032 China

## Abstract

Disrupted Wnt signaling in osteoblastic-lineage cells leads to bone formation defect in osteoporosis. However, the factors repressing Wnt signaling are unclear. In our study, we found that Wnt signaling was suppressed persistently in bone marrow-derived mesenchymal stem cells (BMSCs) during osteoporosis. Accordingly, histone acetylation levels on *Wnt* genes (*Wnt1*, *Wnt6*, *Wnt10a*, and *Wnt10b*) were declined in BMSCs from OVX mice. By screening the family of histone acetyltransferase, we identified that GCN5 expression increased during osteogenic differentiation of BMSCs, whereas decreased after osteoporosis. Further analysis revealed that GCN5 promoted osteogenic differentiation of BMSCs by increasing acetylation on histone 3 lysine 9 loci on the promoters of Wnt genes. Reduced GCN5 expression suppressed Wnt signaling, resulting in osteogenic defect of BMSCs from OVX mice. Moreover, restoring GCN5 levels recovered BMSC osteogenic differentiation, and attenuated bone loss in OVX mice. Taken together, our study demonstrated that disrupted histone acetylation modification in BMSCs lead to bone formation defect during osteoporosis. The findings also introduced a novel therapeutic target for osteoporosis.

## Introduction

Osteoporosis is a common degenerative bone disease in aged population and postmenopausal females^1^. It is characterized by decreased bone density and bone microarchitecture destruction^2^. The fundamental mechanism of osteoporosis is the imbalance between bone formation and resorption^3^. Bone marrow-derived mesenchymal stem cells (BMSCs), which are precursors of osteoblastic-lineage cells, play crucial role in bone formation^4^. Recent studies of our group and others revealed that osteogenic differentiation capacity of BMSCs was impaired during osteoporosis^5–8^. Recovering the osteogenic capacities of BMSCs could reduce bone loss in osteoporosis^7,9–11^, suggesting potential therapeutic strategy for osteoporosis. Understanding the mechanisms of BMSCs dysfunction has become a critical issue in osteoporosis research.

Wnt signaling pathway, a pathway essential for skeletal development and homeostasis^12,13^, play a key role in regulating BMSCs differentiation. Canonical Wnt pathway could activate the expression of Runt-related transcription factor 2 (RUNX2) to promote osteogenic differentiation of BMSCs. Importantly, recent publications have reported that Wnt signaling was impaired in BMSCs of OVX mice^14^. Rescuing Wnt genes expression could restore osteogenic differentiation capacity of BMSCs and attenuated bone loss^15,16^. However, the molecular mechanisms of Wnt signaling repression in osteoporosis is poorly understood.

Emerging evidences revealed that the activation and repression of signaling pathways are controlled by histone acetylation modification^17,18^, a fundamental epigenetic mechanism. Histone acetylation is controlled by the balance between histone acetyltransferases (HATs) and histone deacetylases (HDACs)^19^. Recently, the role of HDACs in Wnt pathway regulation is extensively studied. For example, knockout of HDAC1, HDAC2, or HDAC3 impairs canonical Wnt signaling pathway^17,18^. HDAC1 could regulate β-catenin gene expression by binding to its promoter^20^. HDAC6 suppresses β-catenin acetylation and degradation^21^. On the other side, although HATs contribute to the precise regulation of gene expression^19,22^, the function of HATs in Wnt pathway remains elusive.

In this study, we hypothesized that the dysregulation of Wnt signaling in BMSCs derived from OVX mice is related with histone acetylation modification. By screening the family of HATs, we tried to find out the HATs that affect Wnt signaling pathway and contribute to osteoporosis.

## Results

### Wnt signaling in BMSCs is permanently inhibited during osteoporosis

**Fig. 1:**
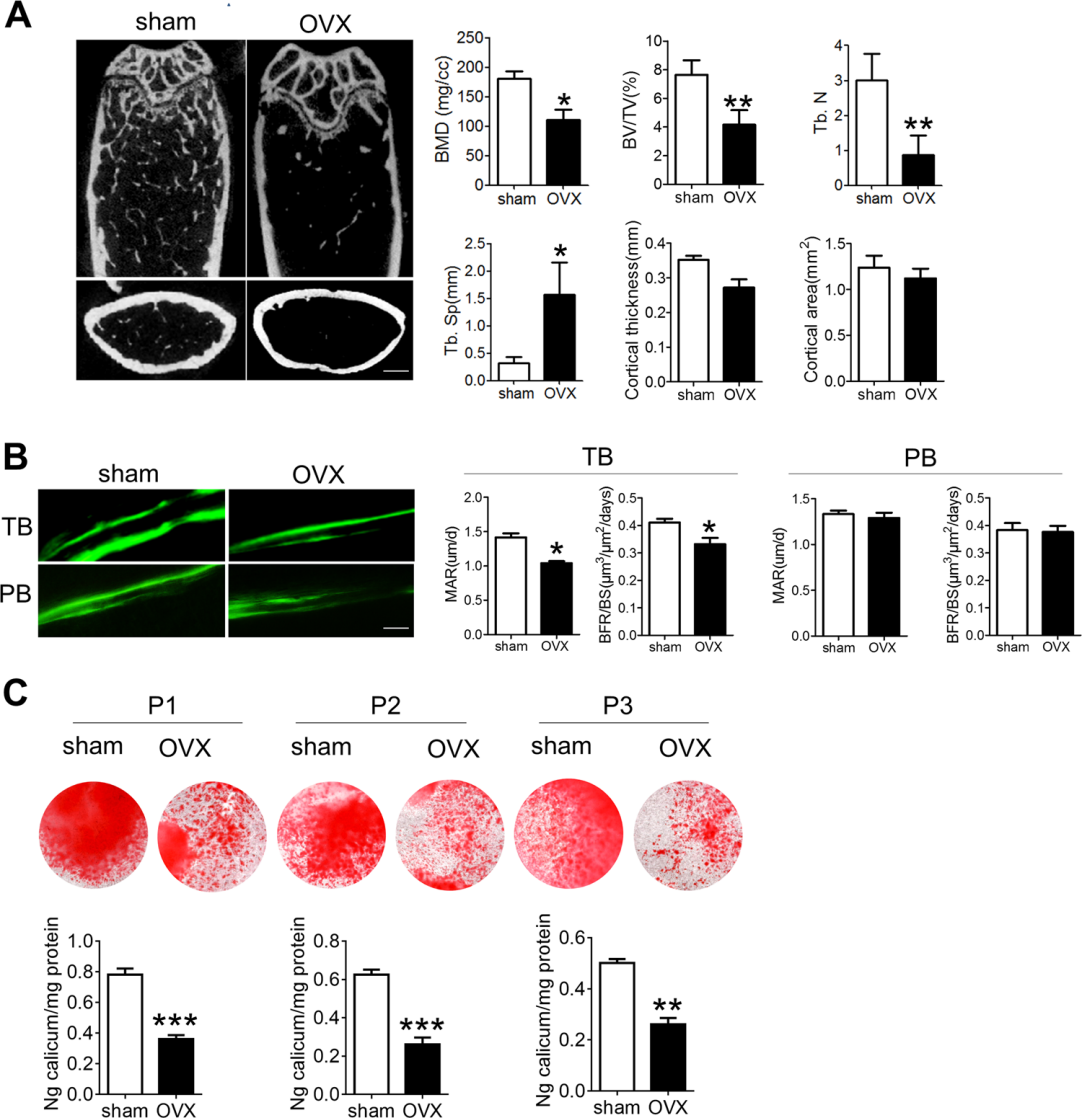
Osteogenic differentiation of BMSCs declined during osteoporosis. **a** Representative micro-CT images of distal femur of sham and OVX mice 2 months after OVX surgery. The structural parameters including bone mineral density (BMD), bone volume/total volume (BV/TV), trabecular bone number (Tb. N), trabecular separation (Tb. Sp), cortical thickness and cortical area were measured. Scale bar: 1 mm (*n* = 4 mice for each group). **b** Representative images of calcein double labeling in trabecular bone (TB) and periosteal bone (PB) of sham and OVX mice. The mineral apposition rate (MAR) and bone formation rate per unit of bone surface (BFR/BS) in trabecular bone and periosteal bone was quantified using image-pro plus software. Scale bar: 50 μm (*n* = 4 mice for each group). **c** Alizarin red staining showed the mineralized nodules formed by sham and OVX BMSCs. Quantification was performed using cetylpyridinium chloride (*n* = 3 cultures for each group). In **a**–**c**, data are presented as mean ± SD. *P < 0.05, **P < 0.01, ***P < 0.001 (Student’s *t*-test)

We established an osteoporosis mice model by ovariectomy (OVX). Two months after OVX surgery, bone mass and bone formation were obviously decreased in osteoporotic femur, compared to sham-operated (sham) mice (Fig. 1a, b). The osteogenic differentiation capacity of BMSCs derived from OVX mice (OVX BMSCs) was significantly decreased, compared with Sham BMSCs (Fig. 1c, Supplementary Fig. S1). It has been demonstrated that disrupted hormones and cytokines in microenvironment lead to the dysfunction of osteoblastic-lineage cells. However, we found that the osteogenic defect of OVX BMSCs is maintained after several passages of in vitro culture (Fig. 1c), suggesting permanent changes in molecular signaling regulation.

Previous reports showed that decreased levels of Wnt signaling, a key pathway regulating osteogenesis, led to bone formation defect of BMSCs during osteoporosis^12,23^. As reported, Wnt/β-catenin signaling was activated during osteogenesis (Supplementary Fig. S2A, S2B). Activating Wnt pathway by Wnt3a promoted osteogenic differentiation, while repressing Wnt signaling by DKK1 inhibited osteogenic differentiation of BMSCs (Supplementary Fig. S2C–S2F).

**Fig. 2:**
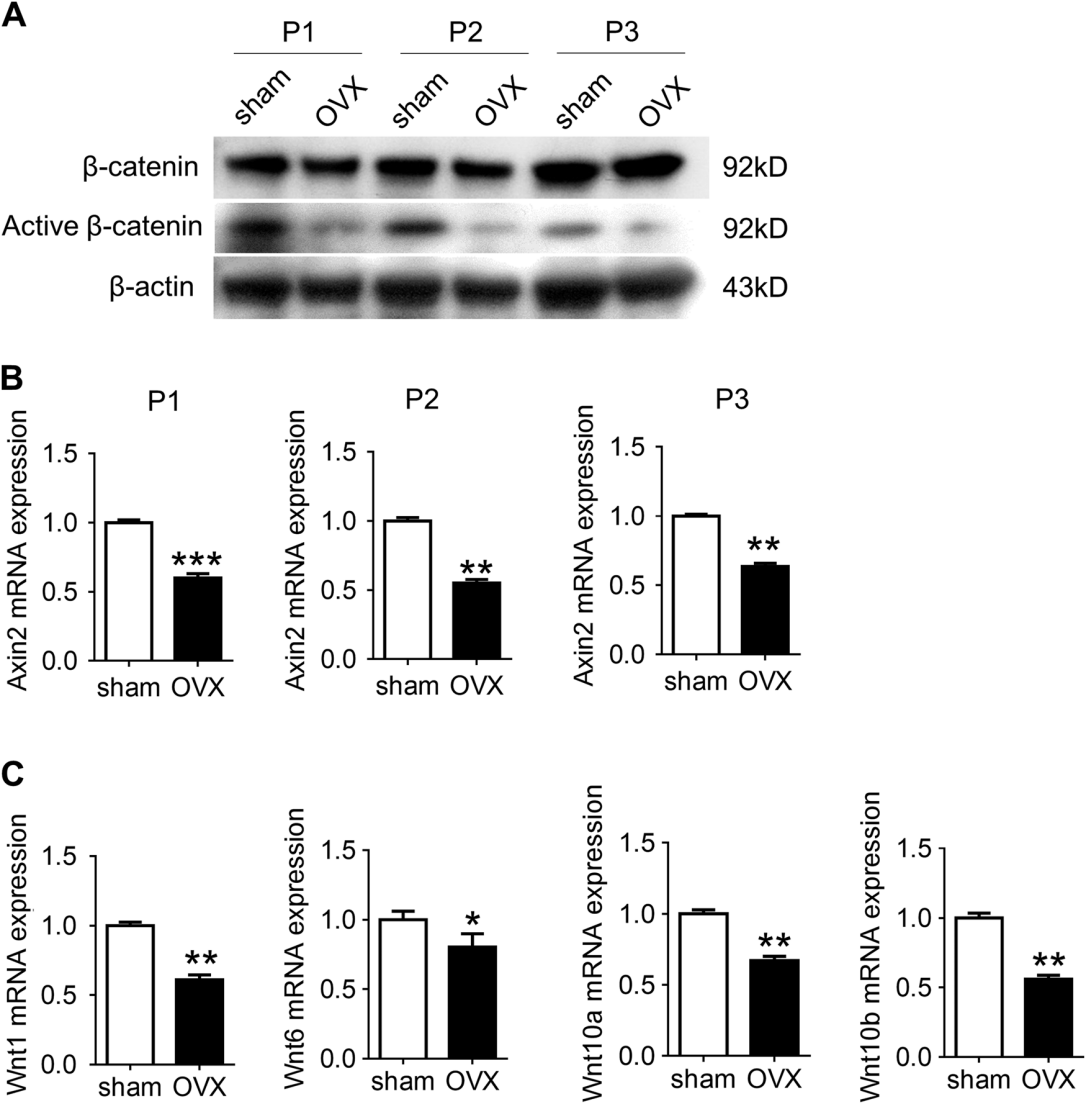
Wnt/β-catenin signaling is persistently suppressed in BMSCs of OVX mice. **a** Protein levels of active β-catenin and β-catenin in sham and OVX BMSCs were measured by western blot assay (*n* = 3 cultures for each group). **b** mRNA levels of Axin2 in sham and OVX BMSCs were measured by real-time RT-PCR (*n* = 3 cultures for each group). **c** mRNA levels of Wnt1, Wnt6, Wnt10a, and Wnt10b in sham and OVX BMSCs were measured by real-time RT-PCR (*n* = 3 cultures for each group). In **b**, **c** data are presented as mean ± SD. *P < 0.05, **P < 0.01, ***P < 0.001 (Student’s*t*-test)

We then compared Wnt signaling in sham and OVX BMSCs. Western blotting revealed that active beta-catenin levels in OVX BMSCs were lower than that in sham BMSCs (Fig. 2a). Real-time reverse transcription (RT)-PCR conformed that the expression of Axin2, a definite target of the Wnt/β-signaling, was decreased in OVX BMSCs, compared with Sham BMSCs (Fig. 2b). The expression of Wnt genes (Wnt1, Wnt6, Wnt10a, and Wnt10b) was also declined in OVX BMSCs (Fig. 2c).

### Histone acetyltransferase GCN5 is suppressed in OVX BMSCs

**Fig. 3:**
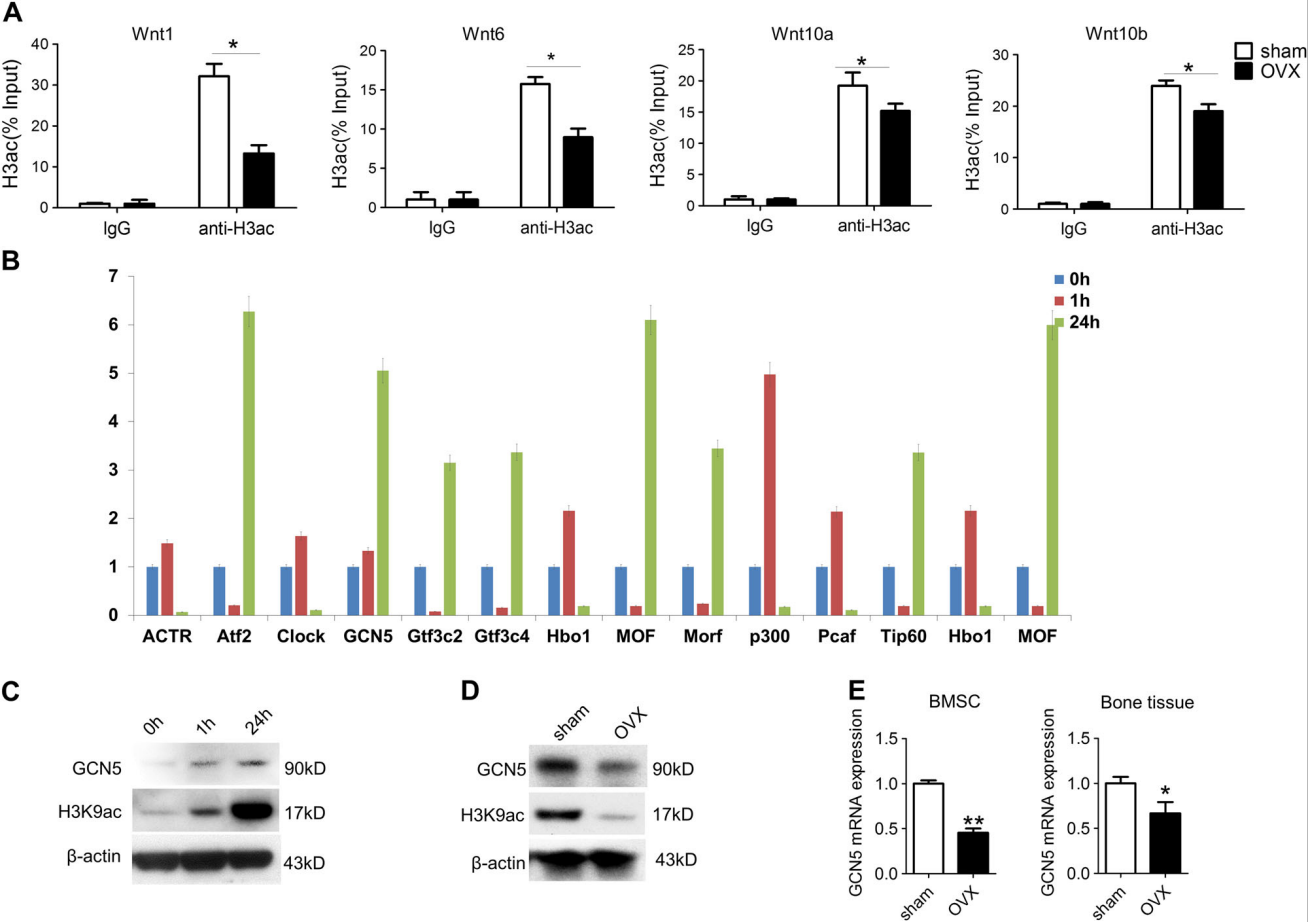
GCN5 expression is declined in BMSCs from OVX mice. **a** ChIP assay was performed to assess the enrichment of H3ac in *Wnt1*, *Wnt6*, *Wnt10a*, and *Wnt10b* promoters in sham and OVX BMSCs. (*n* = 3 cultures for each group). **b** mRNA levels of histone acetyltransferases in BMSCs during osteogenic differentiation were assessed by real-time RT-PCR. The values were normalized to that of β-actin (*n* = 3 cultures for each group). **c** Protein levels of GCN5 and H3K9ac in BMSCs during osteogenic differentiation were assessed by western blot assay. The values were normalized to that of β-actin (*n* = 3 cultures for each group). **d** Protein levels of GCN5 and H3K9ac in sham and OVX BMSCs were examined by western blot assay (*n* = 3 cultures for each group). **e** The mRNA levels of GCN5 in BMSCs and bone tissue derived from sham or OVX mice were assessed by real-time RT-PCR. The values were normalized to that of β-actin. (*n* = 3 cultures for each group)

Histone acetylation modification, a key mechanism of epigenetic regulation, is closely related with the activation or suppression of Wnt signaling in tumor cells and hematopoietic stem cells^24,25^. As expected, CHIP analysis confirmed that the acetylation modifications of histone H3 on the promoters of Wnt genes was decreased in OVX BMSCs (Fig. 3a).

Histone acetylation modification is controlled by deacetylases and acetyltransferases. It has been suggested that histone acetyltransferases play a role in bone development and diseases^26,27^. Therefore, we focused on HATs to elucidate the molecular mechanisms.

Firstly, we identified the HATs controlling osteogenesis. Real-time RT-PCR was performed to screen HATs expression during osteogenic differentiation of BMSCs. Among 14 HATs, only GCN5 increased persistently after 1 h and 24 h of osteogenic induction (Fig. 3b). Western blotting confirmed the increase of GCN5 protein in BMSC during osteogenic differentiation (Fig. 3c). Accordingly, the acetylation levels of H3K9, a major target site of GCN5, were also elevated after BMSC differentiation (Fig. 3c).

We then compared GCN5 as well as H3K9ac levels in sham and OVX BMSCs. Western blotting confirmed GCN5 and H3K9ac were decreased in OVX BMSCs (Fig. 3d). Moreover, GCN5 expression was decreased at the early stage of osteoporosis (Fig. 3e), suggesting that GCN5 is closely related with the dysfunction of OVX BMSCs.

### Decreased GCN5 leads to osteogenic differentiation defect of BMSCs from OVX mice

**Fig. 4:**
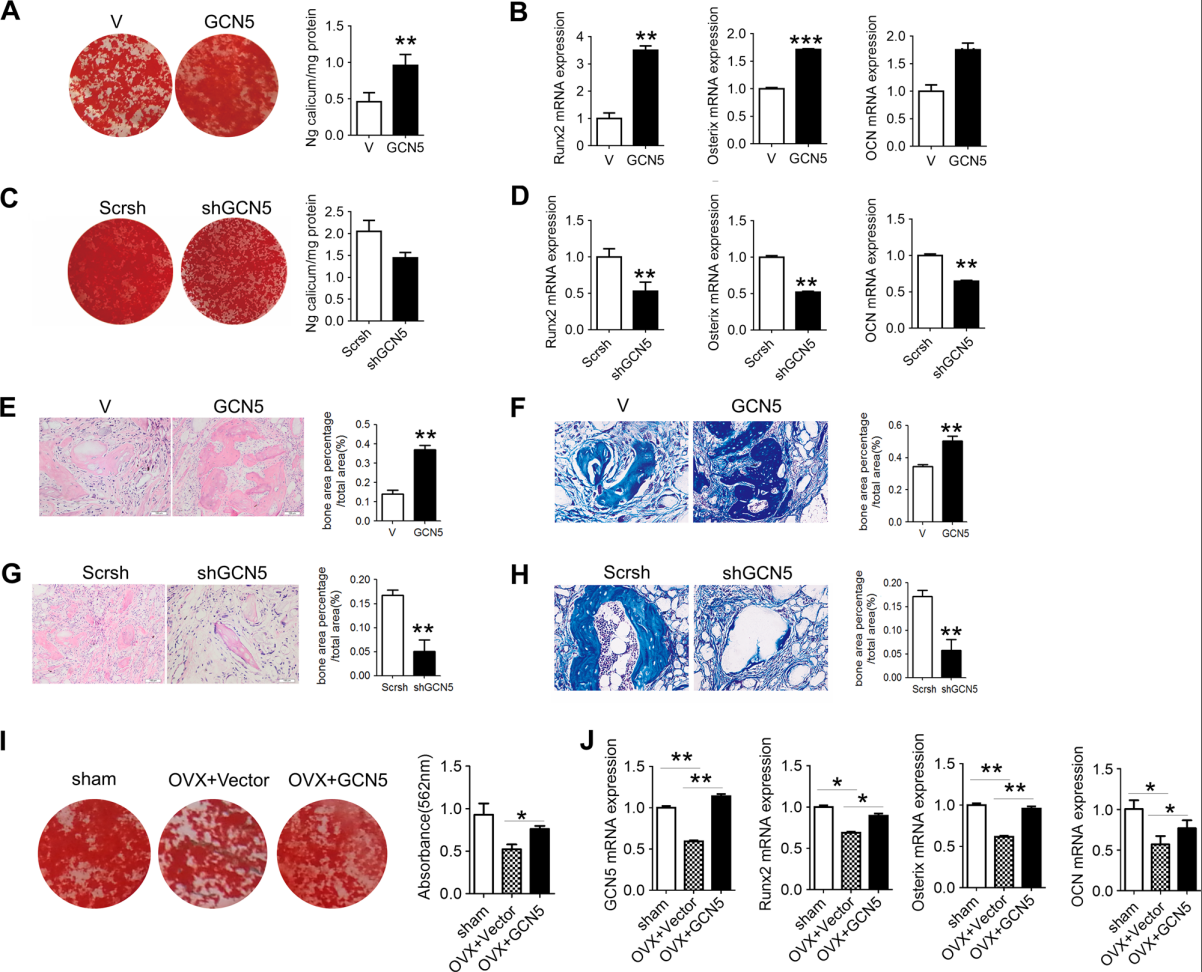
GCN5 promotes osteogenic capacities of BMSCs in vitro and in vivo. **a**, **c** Alizarin red staining revealed the mineralized nodules formed by BMSCs overexpressed (**a**) or knocked-down (**c**) of GCN5 (*n* = 3 cultures for each group). **b**, **d** Real-time RT-PCR assessed the mRNA levels of Runx2, Osterix, and OCN in BMSCs overexpressed (**b**) or knocked-down (**d**) of GCN5 (*n* = 3 cultures for each group). **e**, **g**,** h**, and **e** staining of subcutaneous ectopic osteogenic differentiation of BMSCs overexpressed (**e**) or knocked-down (**g**) of GCN5. The bone area was quantified using image-pro plus software (*n* = 4 cultures for each group). **f**, **h** Masson’s trichrome staining of subcutaneous ectopic osteogenic differentiation of BMSCs overexpressed (**f**) or knocked-down (**h**) of GCN5. The bone area was quantified using image-pro plus software (*n* = 4 cultures for each group). **i** Mineralized nodules formation of sham BMSCs or OVX BMSCs transfected with lentivirus-expressing GCN5 or vector were revealed by Alizarin red staining. Quantification was performed using cetylpyridinium chloride (*n* = 3 cultures for each group). **j** mRNA levels of GCN5 and osteogenic markers, including Runx2, Osterix, and OCN in sham and OVX BMSCs transfected with lentivirus-expressing GCN5 or vector were assessed by real-time RT-PCR. The values were normalized to that of β-actin (*n* = 3 cultures for each group). In **a**–**j** data are presented as mean ± SD. *P < 0.05, **P < 0.01, ***P < 0.001 (Student’s *t*-test)

GCN5 has been found to play crucial roles in development and differentiation^26-30^. However, whether GCN5 is related with osteoporosis is not fully understood. First, we performed gain- and loss-of-function assays to confirm the role of GCN5 in osteogenic differentiation of BMSCs. Fourteen days after osteogenic induction, Alizarin red staining and real-time RT-PCR showed that overexpression of *Gcn5* promoted osteogenic differentiation of BMSCs (Fig. 4a, b), while knockdown of *Gcn5* inhibited osteogenic differentiation of BMSCs (Fig. 4c, d). To verify the in vitro findings, BMSCs overexpressed or knocked-down of *Gcn5* were transplanted subcutaneously into nude mice. After 8 weeks, histological analysis revealed that overexpression of *Gcn5* promoted bone formation of BMSCs (Fig. 4e, f), while knockdown of *Gcn5* inhibited osteogenic differentiation of BMSCs in vivo (Fig. 4g, h).

To explore whether declined GCN5 leads to differentiation defect of BMSCs, we restored GCN5 expression in OVX BMSCs. As expected, transfection of GCN5-lentiviral expression vector largely recovered the osteogenic capacity of OVX BMSCs (Fig. 4i, j).

### GCN5 regulates osteogenesis through Wnt/β-catenin signaling

**Fig. 5:**
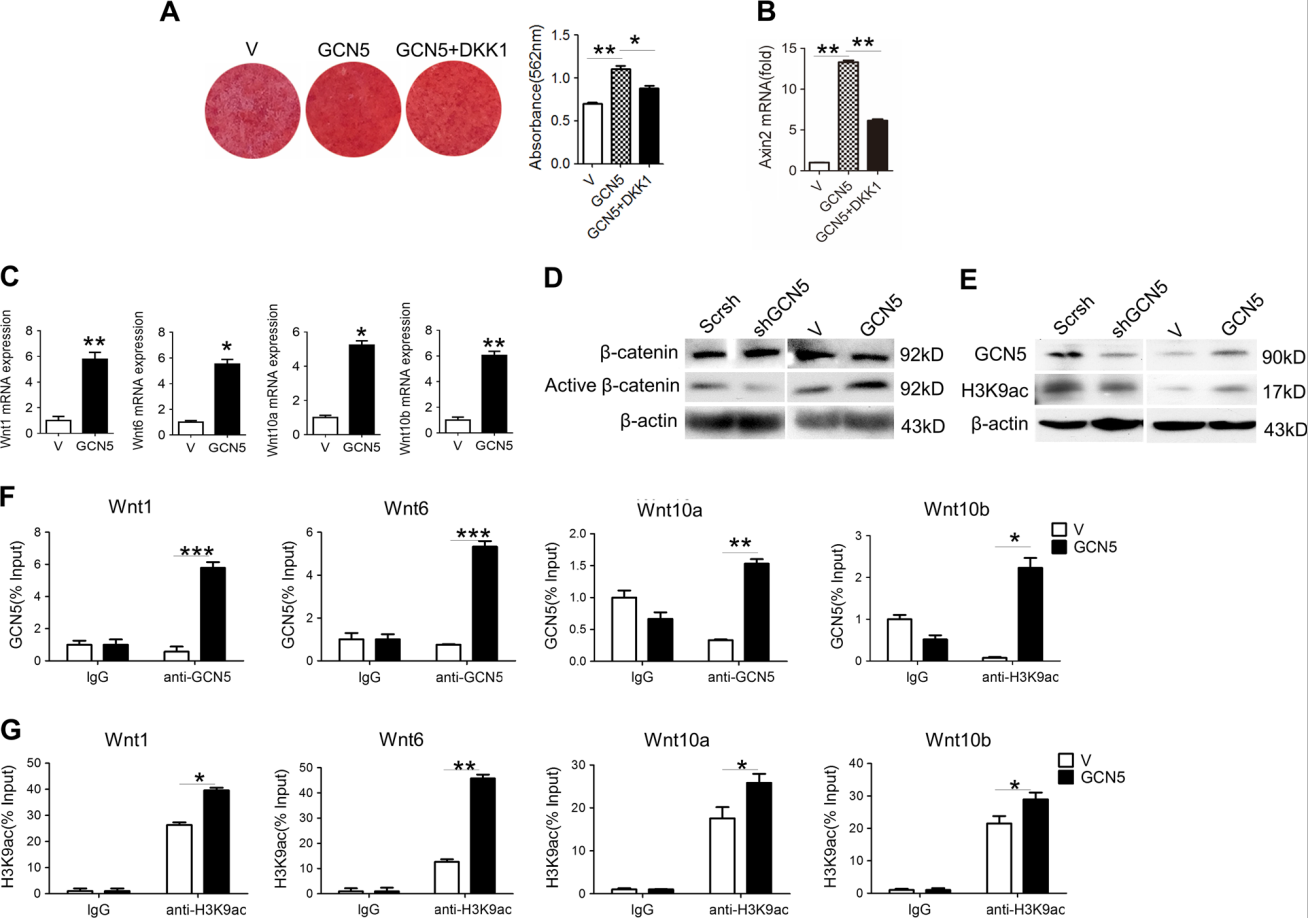
GCN5 enhances transcriptions of *Wnt* genes via increasing acetylation on H3K9 loci. **a** Alizarin red staining revealed mineralized nodules formation in BMSCs transfected with vector or lentivirus-expressing GCN5 with or without DKK1 (*n* = 3 cultures for each group). **b** mRNA level of Axin2 in BMSCs transfected with vector or lentivirus-expressing GCN5 with or without DKK1 (*n* = 3 cultures for each group). **c** mRNA levels of Wnt1, Wnt6, Wnt10a, and Wnt10b in BMSCs transfected with vector or lentivirus-expressing GCN5 were measured by real-time RT-PCR (*n* = 3 cultures for each group). **d** Protein levels of active β-catenin and β-catenin in BMSCs transfected with shRNA of GCN5 or lentivirus-expressing GCN5 were measured by western blot assay (*n* = 3 cultures for each group). **e** Protein levels of GCN5 and H3K9ac in BMSCs transfected with shRNA of GCN5 or lentivirus-expressing GCN5 were measured by western blot assay (*n* = 3 cultures for each group). **f** ChIP assay was performed to assess the binding of GCN5 and mouse IgG to *Wnt1*, *Wnt6*, *Wnt10a*, and *Wnt10b* promoters in BMSCs transfected with vector or lentivirus-expressing GCN5 (*n* = 3 cultures for each group). **g** ChIP assay was performed to assess the enrichment of H3K9ac in *Wnt1*, *Wnt6*, *Wnt10a*, and *Wnt10b* promoters in BMSCs transfected with vector or lentivirus-expressing GCN5 (*n* = 3 cultures for each group). In **a**–**g**, data are presented as mean ± SD. *P < 0.05, **P < 0.01, ***P < 0.001 (Student’s *t*-test)

We next explored whether GCN5 regulates osteogenesis capacity of BMSCs through Wnt pathway. In vitro osteogenesis assay showed that DKK1, an inhibitor of Wnt signaling, abolished the positive effect of GCN5 on osteogenic capacities of BMSCs (Fig. 5a) and activation of Wnt pathway (Fig. 5b). Overexpression of Gcn5 obviously enhanced the expression of these Wnt genes (Fig. 5c). Gain- and loss-of-function assay confirmed that knockdown of GCN5 inhibited the activation of β-catenin, while overexpression of GCN5 promoted β-catenin activation (Fig. 5d).

To identify whether GCN5 regulates Wnt signaling directly through epigenetic modulation, we assessed the histone acetylation modification. Western blot assay showed that H3K9ac levels in BMSCs were increased by GCN5 overexpression, whereas were repressed by knockdown of GCN5 (Fig. 5e). Accordingly, CHIP assay confirmed that overexpression of GCN5 increased the recruitments of GCN5 to the promoter regions of *Wnt1*, *Wnt6*, *Wnt10a*, and *Wnt10b* (Fig. 5f), and enhanced acetylation levels of H3K9ac on those promoters (Fig. 5g). These evidences indicated that GCN5 promoted *Wnt* genes transcriptions by increasing H3K9ac levels on their promoter regions.

### Overexpression of GCN5 attenuates bone mass loss in OVX mice

**Fig. 6:**
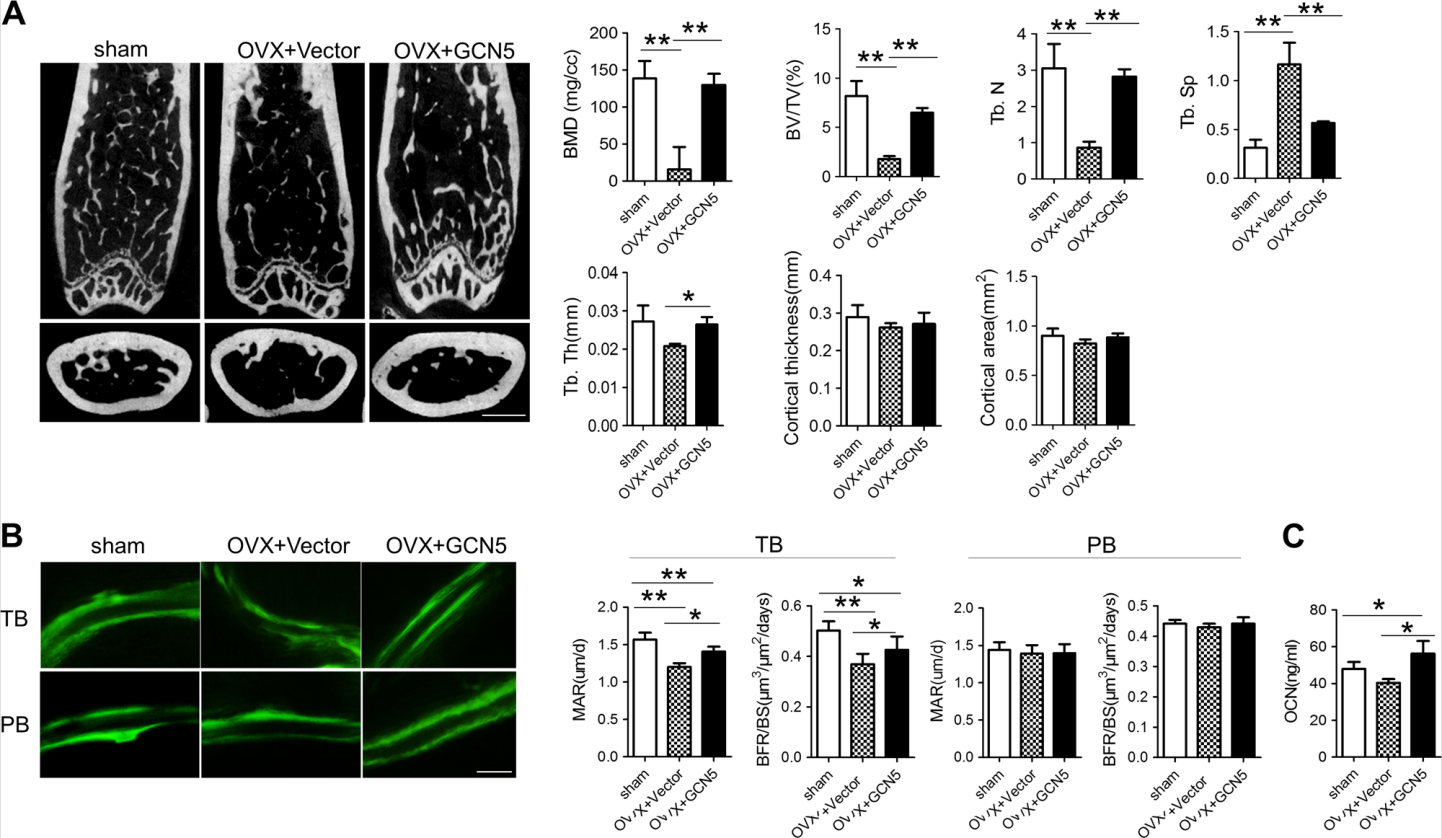
Intra-femoral injection of lentiviral-expressing GCN5 or vector prevents bone mass loss in OVX mice. **a** Representative micro-CT images of distal femur. The structural parameters were calculated. Scale bar: 1 mm (*n* = 4 mice for each group). **b** Representative images of calcein double labeling of trabecular bone (TB) and periosteal bone (PB). The mineral apposition rate (MAR) and bone formation rate per unit of bone surface (BFR/BS) in trabecular bone and periosteal bone was quantified by Image-pro plus software. Scale bar: 40 μm (*n* = 4 mice for each group). **c** Concentration of OCN in bone marrow supernatant was measured by ELISA assay (*n* = 4 mice for each group). In **a**–**c**, data are presented as mean ± SD. *P < 0.05, **P < 0.01, ***P < 0.001 (Student’s *t*-test)

To further verify that recovering GCN5 expression could restore osteogenesis in vivo, we injected either empty vector or *Gcn5-* lentiviral expression vector into mouse femur bone marrow one week after OVX surgery. Six weeks after injection, micro-computed tomography (CT) scan showed that *Gcn5*-lentiviral vector administration largely prevented the bone mineral density (BMD) decline and trabecular bone loss in femurs after OVX (Fig. 6a). To confirm whether GCN5 prevented bone loss by enhancing bone formation, we performed calcein double labeling assay and found that injection of *Gcn5*-lentiviral vector attenuated the decline of bone formation in trabecular bone, but not cortical bone (Fig. 6b). The concentration of OCN, a biomarker of bone formation, was also enhanced in bone marrow after *Gcn5* overexpression (Fig. 6c). Taken together, these results indicated that recovering GCN5 expression rescued bone formation in OVX mice.

### Overexpression of GCN5 rescues osteogenic differentiation of endogenous BMSCs in OVX mice

To investigate whether Gcn5-lentiviral vector injection recovered osteogenesis capacity of BMSCs, we isolated BMSCs from OVX mice 6 weeks after the lentivirus administration.

**Fig. 7:**
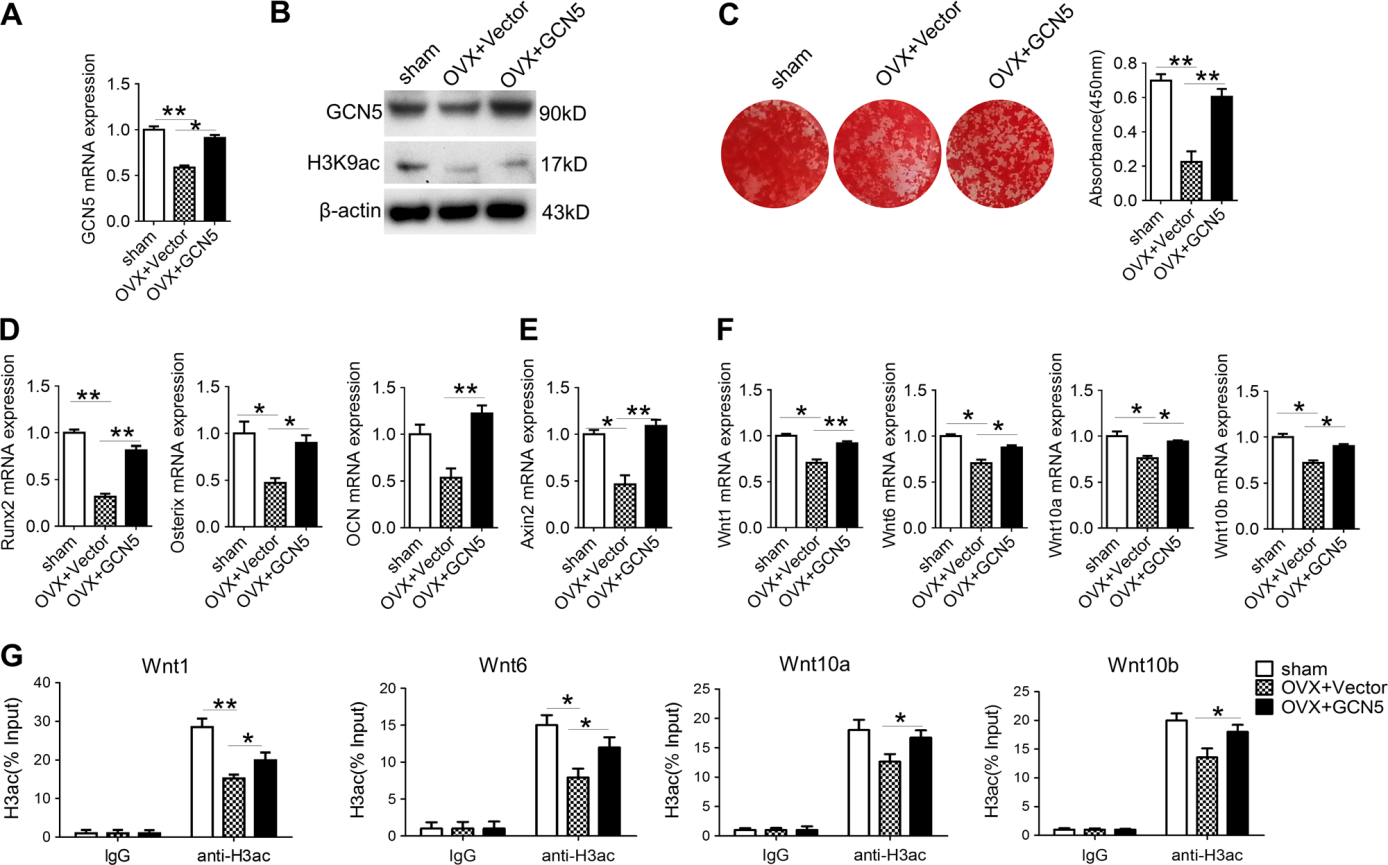
Intra-femoral injection of lentiviral-expressing GCN5 restores osteogenic differentiation potential of endogenous BMSCs. **a** mRNA levels of GCN5 in BMSCs from sham or OVX mice received injection of lentivirus or vectors were measured by real-time RT-PCR (*n* = 4 cultures for each group). **b** Protein levels of GCN5 and H3K9ac in BMSCs from sham or OVX mice received injection of lentivirus or vectors were measured by western blot assay (*n* = 4 cultures for each group). **c** Alizarin red staining revealed the mineralized nodules formed by BMSCs (*n* = 4 cultures for each group). **d**, **e** mRNA levels of *Runx2*, *Osterix*, *OCN* (**d**), and *Axin2* (**e**) in BMSCs were measured by real-time RT-PCR (*n* = 4 cultures for each group). **f** mRNA levels of *Wnt1*, *Wnt6*, *Wnt10a*, and *Wnt10b* in BMSCs were measured by real-time RT-PCR (*n* = 4 cultures for each group). **g** ChIP assay was performed to assess the enrichment of H3ac in *Wnt1*, *Wnt6*, *Wnt10a*, and *Wnt10b* promoters in BMSCs (*n* = 3 cultures for each group). In **a**–**g**, data are presented as mean ± SD. *P < 0.05, **P < 0.01 (Student’s *t*-test)

Real-time RT-PCR and western blot assay revealed that *Gcn5*-lentivirus injection enhanced both mRNA and protein levels of GCN5 in OVX BMSCs (Fig. 7a, b). Consistently, the protein level of H3K9ac in OVX BMSCs was also increased by *Gcn5*-lentivirus injection (Fig. 7b). Osteogenesis assay showed that overexpression of GCN5 largely recovered the osteogenic differentiation of OVX BMSCs (Fig. 7c, d). Consistent with the findings that GCN5 functions through Wnt pathway, the mRNA levels of *Axin2* and *Wnt* genes were also enhanced in BMSCs after *Gcn5*-lentiviral vector injection (Fig. 7e, f). Consistently, H3ac levels in promoter regions of *Wnt* genes were also increased after overexpression of GCN5 (Fig. 7g). Taken together, the results demonstrated that local administration of *Gcn5*-lentiviral vector activated Wnt signaling to promote osteogenic differentiation of endogenous BMSCs.

## Discussion

Dysfunction of BMSCs lead to bone formation defect in osteoporosis^7,8,10,11,31,32^. But its mechanism remains elusive. In this study, we found that an epigenetic mechanism that represses Wnt signaling to inhibit osteogenic differentiation of BMSCs during postmenopausal osteoporosis. The decrease of histone acetylation modification lead to persistent repression of Wnt signaling. By restoring histone acetylation levels in OVX BMSCs, the osteogenic capacities of BMSCs was recovered in osteoporotic mice. Our study revealed a novel mechanism of BMSCs dysfunction during osteoporosis and introduces a novel insight into the prevention of osteoporosis.

Our findings indicated that Wnt signaling is precisely modulated by histone acetylation. In this study, we found that acetylation on histone declined in OVX MSCs. Histone acetylation lost the condensed chromatin and enhances gene transcription. Reduced histone acetylation limited the transcription of genes regulating BMSC functions. Consistently, the Wnt/β-catenin signaling, one of the crucial pathway for osteogenesis, was also repressed in OVX BMSCs. Restoring histone acetylation levels by GCN5 recovered expression of Wnt genes in OVX BMSCs, suggesting the crucial role of histone acetylation in controlling Wnt pathway. Besides acetylation, histone could be modified by various modifications. Further studies focused on histone epigenetic modifications would expand our understanding of the molecular mechanism of osteoporosis.

Previous studies majorly focused on the function of HDACs in Wnt signaling^18,20,21^. However, the role of HATs in Wnt signaling regulation remains elusive. By analyzing the family of HATs, we found that GCN5 played important roles in Wnt signaling regulation and BMSCs osteogenesis. Moreover, GCN5 controlled the expression of Wnt ligands through histone acetylation modification. Since Wnt signaling is one of the most important pathway involved in development, regeneration and diseases, it is interesting to investigate whether GCN5 play other physiological or pathological roles by regulating Wnt signaling.

By injecting lentivirus-expressing GCN5 into femur of OVX mice, we restored bone volume and density in osteoporotic mice. After isolation of BMSCs from bone marrow, we confirmed that injection of GCN5 lentivirus recovered osteogenic capacity of OVX BMSCs, indicating that GCN5 recovered bone mass by regulating BMSCs. Since the lentivirus is not specific to BMSCs, it is possible that cells other than BMSCs, such as osteoclasts and immune cells are also involved in the recovery of osteoporosis. Based on previous reports, GCN5 upregulates T-cell activation^33^ and is required for iNKT cell development^34^. Therefore, conditioned knockout of GCN5 in BMSCs might be needed to explore GCN5 function in vivo.

Emerging studies revealed that GCN5, a highly conservative histone HAT, is involved in various diseases. GCN5 is widely studied in tumors and has been tested as therapeutic targets for cancer. We recently found that GCN5 controlled the pro-angiogenic factors secretion of BMSC through increasing VEGF expression. Taken together with this study, we revealed that GCN5 is a crucial factor regulating osteogenesis during bone remodeling. Importantly, recovering GCN5 expression rescued the dysfunction of BMSCs and attenuated bone loss during osteoporosis, indicating that GCN5 is a potential target for osteoporosis treatment.

## Materials and methods

### Animals

Eighty 8-week female C57BL/6 mice purchased from the Laboratory Animal Research Center of the Fourth Military Medical University were used for cell culture and osteoporosis model establishment. Twenty-two 6-week female immunocompromised nude mice (BALB/c) for subcutaneous transplantation and twenty-two 8-week female C3H mice used for femur injection of lentivirus were purchased from Vital River Laboratory Animal Technology Co. Ltd. (Beijing, China). All protocols have been approved by the Animal Care Committee of the Fourth Military Medical University and have met the NIH guidelines for care and use of laboratory animals in this study.

### BMSCs culture

Murine bone marrow mesenchymal stem cells (BMSCs) were flushed from long bones by using α-MEM medium supplemented with 20% FBS. After centrifuge, BMSCs were seeded into 10-cm dishes with α-MEM (Gibco BRL, Gaithersburg, MD, USA) supplemented with 20% FBS (Sijiqing, Hangzhou, China), 2 mM l-glutamine (Invitrogen, Carlsbad, CA, USA), 100 U/ml penicillin, and 100 mg/ml streptomycin (Invitrogen, Carlsbad, CA, USA). Cells were incubated in a humidified atmosphere of 5% CO_2_ at 37 °C. The culture medium was first changed after 24 h of seeding and followed by change every other day. After confluence, cells were passaged by using 0.25% trypsin. BMSCs at the first passage were used in all experiments.

### Osteogenic differentiation in vitro

In all, 5 × 10^5^ cells were seeded in each well of six-well plates. Then, cells were incubated with osteogenic-inducing medium supplemented with 100 μg/ml ascorbic acid, 2 mmol/l β-glycerophosphate and 10 nmol/l dexamethasone for 14 days. The media was changed every other day.

### Alizarin red staining

The Alizarin red staining was conducted as previously reported^10^. In brief, BMSCs were fixed with 60% isopropanol for 1 min at room temperature after washing with Phosphate-buffered saline(PBS) twice. Then, cells were washed twice by using ddH2O, followed by staining with 1% alizarin red (Sigma-Aldrich, St. Louis, MO, USA) for 10 min at room temperature. The quantification was performed by using 5% cetylpyridinium chloride to elute the staining. The absorbance was determined by a spectrophotometer at the wavelength of 562 nm. In order to exclude the influence of different cell numbers on the quantification, results were normalized to the concentration of protein.

### Colony-forming unit-osteoblast (CFU-OB) assays

The CFU-OB assays were performed as previously described^35^. Briefly, after whole bone marrow cells were flushed from long bones, one million cells were seeded in 6-cm dishes. After 48 h, the cells were incubated in osteogenic-inducing medium for 14 days. The cells were washed by PBS twice, fixed in 60% isopropanol for 1 min, washed twice with ddH2O, and stained with 1% Alizarin red (Sigma-Aldrich, St. Louis, MO, USA) for 10 min at room temperature. Colonies with > 50 cells were counted.

### Lentiviral vector construction and transduction

Wild-type mouse GCN5 was amplified by PCR. *Not*I and *Kpn*I were used to digest the amplicons before inserted into the Pent3c lentiviral vector. The sequences of the primers were: forward: CGGGGTACCATGGCGGAACCTTCCCAGGC; reverse: AAGGAAAAAAGCGGCCGCGGGACCAGGCTCAGGATGCTCA. The primers for GCN5 shRNA were: forward: CCGGGCTACCTACAAAGTCAATTATCTCGAGATAATTGACTTTGTAGGTAGCTTTTTG; reverse: AATTCAAAAAGCTACCTACAAAGTCAATTATCTCGAGATAATTGACTTTGTAGGTAG.

Restriction enzymes *Age*I and *Eco*RI were used to digest the PCR product before incorporated into the PLKO.1 vector (Invitrogen). Sanger sequencing was performed to verify the inserted fragments. To produce the lentivirus, 293T cell line was co-transfected with two packaging vectors (psPAX2 and pMD2.G) and two plasmid vectors. To get rid of the cell debris, supernatant was centrifuged at 1000 rpm for 10 min, followed by filtered through a 0.45-μm polyethersulfone low protein-binding filters. The titer of the lentivirus was 105 IFU/ml, assessed by a quantify kit (Clontech, CA). In all, 8 mg/ml polybrene (Sigma, St. Louis, MO) were used when virus was transfected into BMSCs. A vector inserted with scrambled GCN5 was used as a negative control.

### Subcutaneous bone formation in nude mice

BMSCs were transfected with lentiviral vector expressing either *Gcn5* or shRNA, as well as their negative control vectors. Three days after transfection, 2 × 10^6^ BMSCs were mixed with 15 mg of hydroxyapatite/tricalcium phosphate (HA/TCP) ceramic particles (Sigma-Aldrich) and implanted into the dorsal subcutaneous space of NOD/SCID mice (Fourth Military Medical University Experimental Animal Center). Eight weeks after transplantation, the implants were harvested, fixed with 4% paraformaldehyde (PFA) and decalcified with buffered 10% EDTA (pH 7.4). Histological sections were stained with H&E or Masson’s Trichrome (BaSO Diagnostic Inc, Guangdong, China).

### Intra-femoral injection of lentivirus vectors

The lentivirus-expressing GCN5 and the control vector were concentrated into 5–10*10^5^ transducing units/ml by using the Lenti-X™ Concentrator (Cat. Nos. 631231 & 631232) before the intra-femoral injection. The injection of lentivirus was conducted 1 week after the OVX surgery was performed. After anesthetization, a small incision was made over the kneecap to allow the 22-gauge needle to pierce into the femur. The mice were either injected with 20 ul lentivirus-expressing GCN5 or empty vector. After the injection, the incision was sutured carefully to recover leg movement of the mice. Analgesic drug was added into water after the surgery and feed the mice for 1 week.

### Calcein double labeling assay

The mice were intraperitoneally injected with 15 mg/kg of calcein (Sigma-Aldrich) 15 and 3 days prior to sacrifice. The femora were then fixed in 4% PFA for 24 h and embedded in methylmethacrylate without being decalcified. The samples were sectioned using a Sliding Microtome, and the slices were observed and photographed under a fluorescence microscope. The dynamic bone formation rate was measured by mineral apposition rate (MAR) and bone formation rate per unit of bone surface (BFR/BS). The calculation was performed in areas randomly selected from five fields in distal metaphysis of femur and periosteum.

### Micro-CT analysis of femora

The femora were fixed in 4% phosphate-buffered PFA after the mice were sacrificed. The Locus SP Pre-Clinical Specimen micro-CT system (GE Healthcare, USA) was used to scan the samples with 8-mm resolution, 50 kV tube voltage and a 0.1 mA tube current. The region of interest (ROI) was selected for the area from 0.5 mm to 1.5 mm below the growth plate in the distal ends of femur. The ROI of cortical bone was conducted in the region of the middle of the femur.

### Real-time RT-PCR assay

Total RNA was extracted by TRIzol reagent (Invitrogen Life Technology, Carlsbad, CA). One microgram of total RNA was used for reverse transcription. The process was performed by using a PrimeScript RT reagent kit (Takara Bio, Shiga, Japan). Real-time RT-PCR was conducted by using a Quantitative SYBR Green Kit (Toyobo, Osaka, Japan) and was detected by ABI Prism 7500 Sequence Detection System (Applied Biosystems, Foster City, CA). The Supplementary Table S1 shows the primer sequences. The PCR program was set as: 94 °C, 3 min; 94 °C, 15 s; 60 °C, 30 s, for 40 cycles. *β-actin* was used as control. The results were calculated by ΔΔ−ct method.

### ChIP assay

ChIP assay was performed following manufacturer’s instructions of a ChIP assay kit (Millipore, Catalog 17-371, Billerica, MA). The antibody against GCN5 (Santa Cruz Biotechnology, sc-365321 × ), H3K9ac (Abcam, ab10812) and H3ac (Millipore, 06-599) were used. The mouse IgG and anti-RNA Polymerase II (Millipore) were used as negative control and the positive control, respectively. The precipitated DNA samples were assessed by real-time PCR. Primers were designed from the region of 500-bp distance to the transcription start sites. The primers of Wnt1 were: forward GGTTAGCCTGTCAGCTCTTTGC; reverse TCAGGGCCACAGACAATGAG; The primers for Wnt6 were: forward CTTCCTTCCCCCAAAGAAATG; reverse GTCCAACAGCTCTTCCCTACCTATC; The primers for Wnt10a were: forward TCCTTCCTTCACCCTCTGCAT; reverse TAGTGTCTAAGGGTTCTACCCCAAGT. The primers for Wnt10b were: forward TCCAAAGGAAAGGTTTTAGCCATA; reverse CCCACCCTTCCTGCTGAA. The values of results were normalized to the input value.

### Protein isolation and western blotting

RIPA buffer (Beyotime) with protease inhibitor were used to extract protein from BMSCs. BCA quantitative assay (Beyotime) was used to measure the protein concentration. Twenty microgram protein was separated by 4 – 12% Tris-glycine sodium dodecyl sulfate-polyacrylamide gel (Invitrogen Life Technology), followed by transferred onto a polyvinylidene fluoride(PVDF) membrane (Millipore). Blocking was performed by using 5% BSA for 2 h at room temperature. The membrane was incubated with primary antibodies overnight at 4 °C.

Secondary antibodies (Cowin Biotech Co, Beijing, China) were incubated for 2 h at room temperature. The protein accumulation was detected by Western-light Chemiluminescent Detection System (Peiqing, Shanghai, China). Antibodies against GCN5 (Santa Cruz Biotechnology, sc-365321 × ), H3K9ac (Abcam, ab10812), active β-catenin (Millipore, 05-665), GAPDH (Cowin Biotech, CW0100), and β-actin (Cowin Biotech, CW0096A) were used.

### Statistical analysis

Data were showed as means ± standard deviation (SD). Statistical analyses were used by using the Prism software (GraphPad, La Jolla, CA). Statistical differences were assessed by *t*-test or one-way analysis of variance. *P < *0.05 was considered as significant.

## Electronic supplementary material

supplementary
