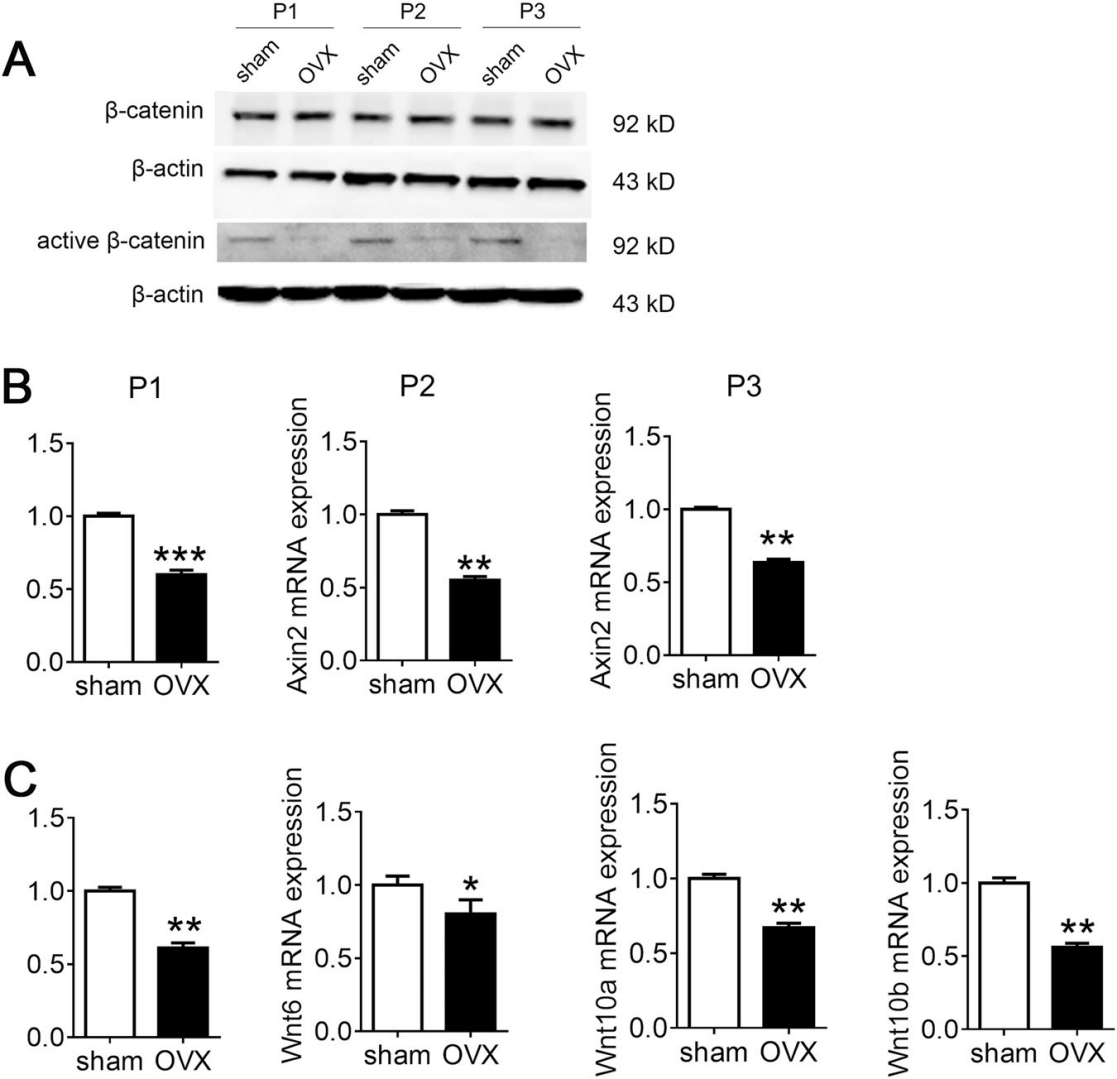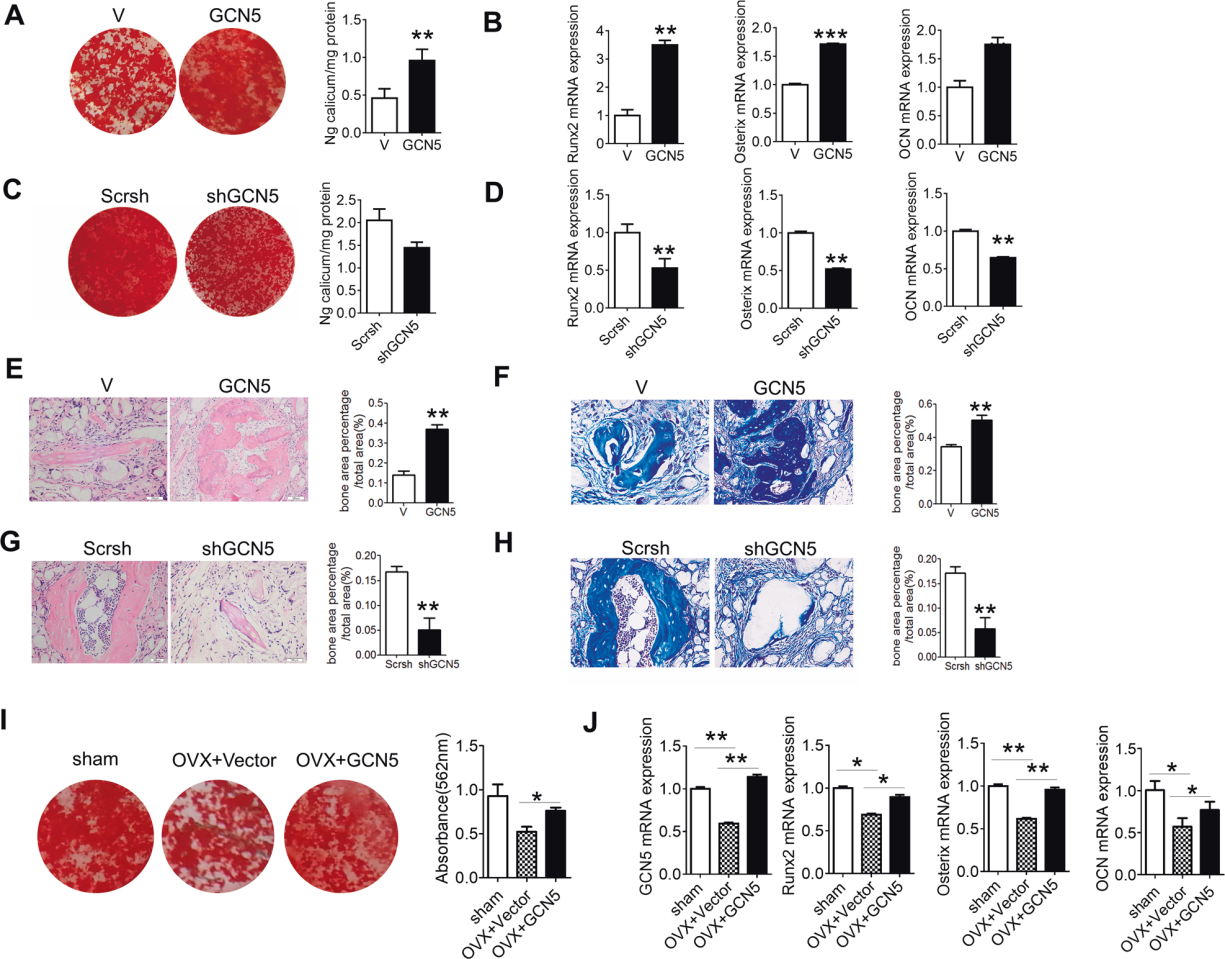# Correction: Epigenetic inhibition of Wnt pathway suppresses osteogenic differentiation of BMSCs during osteoporosis

**DOI:** 10.1038/s41419-022-04616-z

**Published:** 2022-02-28

**Authors:** Huan Jing, Xiaoxia Su, Bo Gao, Yi Shuai, Ji Chen, Zhihong Deng, Li Liao, Yan Jin

**Affiliations:** 1 State Key Laboratory of Military Stomatology & National Clinical Research Center for Oral Diseases & Shaanxi International Joint Research Center for Oral Diseases, Center for Tissue Engineering, School of Stomatology, The Fourth Military Medical University, Xi’an, Shaanxi 710032 China; 2 Xi’an Institute of Tissue Engineering and Regenerative Medicine, Xi’an, Shaanxi 710032 China; 3 Key Laboratory of Shaanxi Province for Craniofacial Precision Medicine Research, College of Stomatology, Xi’an Jiaotong University, Xi’an, Shaanxi 710004 China; 4 Department of Orthopaedic Surgery. Xijing Hospital, Fourth Military Medical University, Xi’an, Shaanxi 710032 China; 5 Department of Oral Implantology, School of Stomatology, State Key Laboratory of Military Stomatology, The Fourth Military Medical University, Xi’an, Shanxi 710032 China; 6 Department of Otolaryngology, Xijing Hospital, Fourth Military Medical University, Xi’an, Shaanxi 710032 China

Correction to: *Cell Death and Disease* 10.1038/s41419-017-0231-0, published online 7 February 2018

The original version of the above article contains errors that need to be corrected. Incorrect images of the Vector group (V) and Scrsh group in Fig. 4E were used in figure assembly and need to be corrected. The Western blotting images in Fig. 2A were also replaced with images from a repeat experiment. The corrected Figs. 2 and 4 are given below.

The correction does not affect the conclusions of the above paper. The authors apologize for the mistakes and any inconvenience caused.