# Supplementary material for: Epigenetic inhibition of Wnt pathway suppresses osteogenic differentiation of BMSCs during osteoporosis
Source: Cell Death Dis. 2018 Feb 7;9(2):176. doi: 10.1038/s41419-017-0231-0 (PMC5833865; doi:10.1038/s41419-017-0231-0)
Supplement: Supplementary file 1 — supplementary [file 41419_2017_231_MOESM1_ESM.docx]

**Supplementary Fig.1**


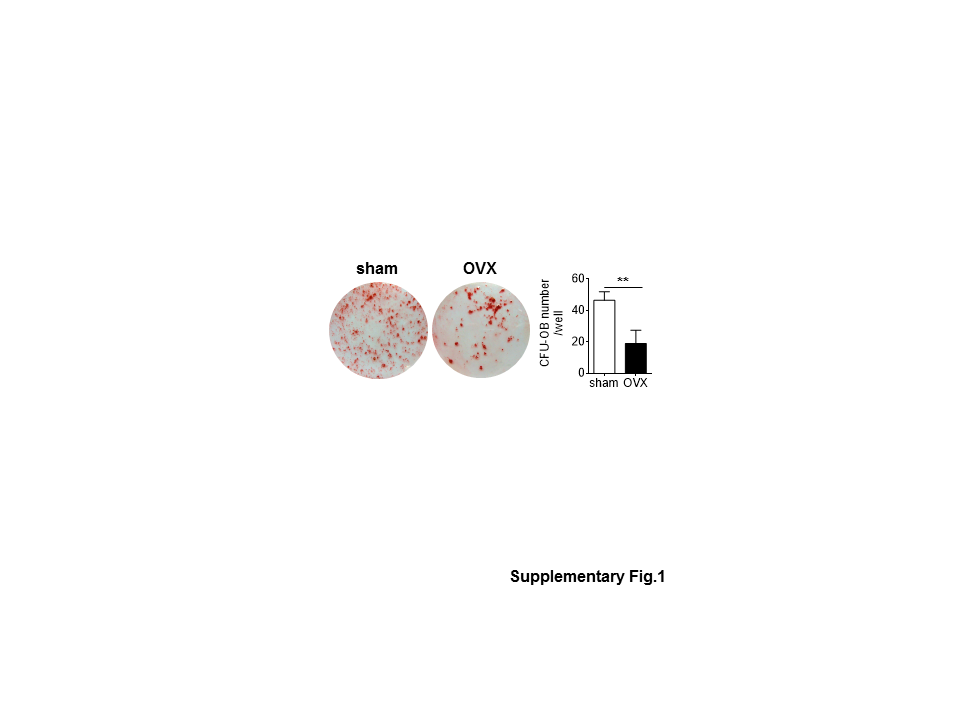


**Supplementary Fig.S1 The osteogenic differentiation of BMSCs is suppressed after osteoporosis.**

The osteoblast numbers in sham and OVX mice were assessed by using Colony-Forming Unit-Osteoblast (CFU-OB) assays (n = 3 cultures for each group).

**Supplementary Fig.2**


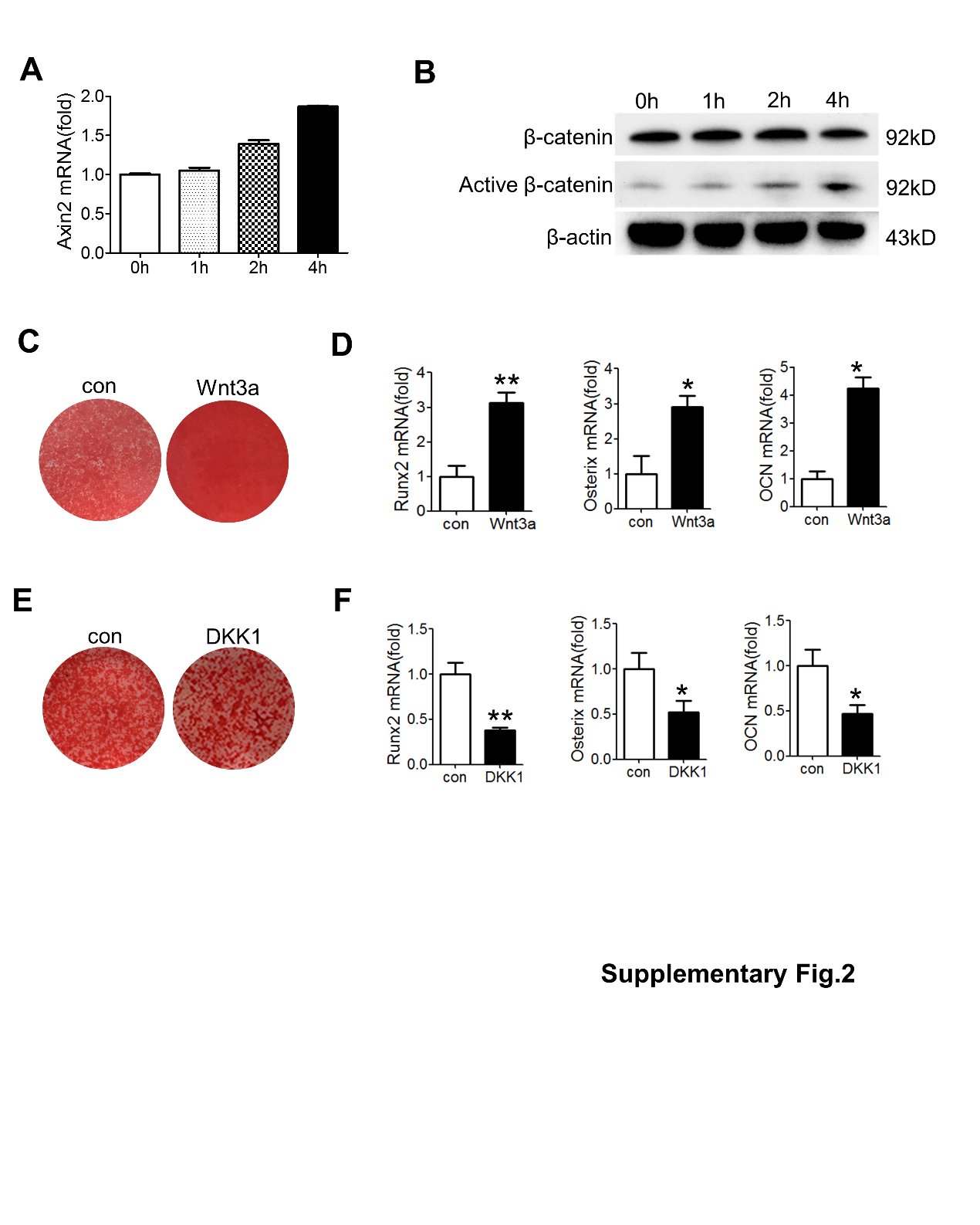


**Supplementary Fig.S2 Wnt signaling is required for osteogenic differentiation of BMSCs.**

A mRNA levels of Axin2 in BMSCs during osteogenic differentiation of BMSCs were measured by Real-time RT-PCR. (n = 3 cultures for each group).

B Protein levels of active β-catenin and β-catenin in BMSCs during osteogenic differentiation of BMSCs were measured by Western blot assay. (n = 3 cultures for each group).

C, E Alizarin red staining revealed mineralized nodules formation in BMSCs treated with Wnt3a (C) or DKK1 (E). (n = 4 cultures for each group).

D, F mRNA levels of Runx2, Osterix and OCN in BMSCs treated with Wnt3a (D) or DKK1 (F) were measured by Real-time RT-PCR. (n = 3 cultures for each group).
